# Trapping Xe in Nanocages Using a Plasma

**DOI:** 10.1002/smsc.202500136

**Published:** 2025-07-11

**Authors:** Laiba Bilal, Asim Khaniya, Dustin Olson, Staci Moulton, Arrelaine Dameron, Xiao Tong, Lynne Ecker, Dario Stacchiola, Jorge Anibal Boscoboinik

**Affiliations:** ^1^ Electrical and Computer Engineering Department Stony Brook University Stony Brook NY 11790 USA; ^2^ Center for Functional Nanomaterials Brookhaven National Laboratory Upton NY 11973 USA; ^3^ Tritium Technology Division Savannah River National Laboratory Aiken SC 29808 USA; ^4^ Forge Nano, Inc. Thornton CO 80241 USA; ^5^ Nuclear Science and Technology Department Brookhaven National Laboratory Upton NY 11973 USA

**Keywords:** atom trapping, confinement, nanocages, noble gases, plasma, silicate, xenon

## Abstract

Xenon (Xe), the largest noble gas with nonradioactive isotopes, is difficult to contain in a solid matrix. This study demonstrates the trapping of Xe single atoms within subnanometer‐sized silicate nanocages (NC) supported on metal powders. The Xe gas is first ionized using a plasma. The ions then enter the NC and get trapped in the solid upon gaining an electron from the metal support. This work presents the first demonstration of room‐temperature trapping of Xe atoms in a high‐surface‐area material using a simple ionization method. This can lead to various practical applications such as Xe separation from air, improving the efficiency and safety of nuclear reactors, aiding in nuclear nonproliferation efforts, and producing medical isotopes, among many others.

## Graphical abstract

Silicate nanocages supported on metal powders trap xenon (Xe) atoms when exposed to a Xe plasma. Three major advances are achieved in this work including trapping Xe in (1) high‐surface‐area powder materials, using (2) a nonprecious metal support, and (3) a simple ionization method.

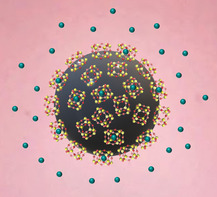

## 1 Introduction

Room‐temperature trapping of xenon (Xe) gas atoms in silica nanocages (NC) supported on metal powders of ruthenium (Ru) and cobalt (Co) is investigated by X‐ray photoelectron spectroscopy (XPS). Xe, being a noble gas, has a very low reactivity ^[^ ^1^ ^]^ and it is difficult to immobilize in a solid matrix. This gas is used in many applications. It was first utilized in flash lamps for photography^[^ ^2^ ^]^ and continues to serve this function due to its ability to produce a bright, white flash when excited. Its applications extend across various industries, showcasing its versatility. In entertainment and technology, Xe is crucial in plasma televisions and cinema projectors, enhancing the quality of visuals.^[^ ^3^ ^]^ Xe is also the most used propellant in ion propulsion systems for spacecraft^[^ ^4^ ^]^ through thrusters, leveraging its inert nature and ionization efficiency. In the medical industry,^[^ ^5^ ^]^ Xe is valued for its anesthetic properties^[^ ^6^ ^]^ that offer safety for sensitive patients and is used as an agent in medical imaging^[^ ^7^ ^]^ to enhance diagnostic procedures without biochemical interactions. In semiconductor manufacturing, Xe is instrumental in lithography^[^ ^8^ ^]^ for creating integrated circuits, where its plasma generates extreme ultraviolet light for etching fine details onto silicon wafers. Recently, Xe's potential in skincare has been explored, with studies suggesting that it can aid in skin repair,^[^ ^9^ ^]^ leading to its inclusion in some dermatological products. These diverse applications underscore Xe's unique properties and importance across various scientific and industrial fields.

Despite its extensive usage, Xe is rare on earth, occurring at only one part per ten million by volume in dry air.^[^ ^2^ ^]^ Like several other noble gases, Xe is found in meteorites and is typically obtained on a small scale through the fractional distillation of liquid air.^[^ ^10^ ^]^ Xe's concentration in the earth's crust and the atmosphere is much lower than predicted; this is often referred to as “missing xenon paradox.”^[^ ^11^ ^]^ This scarcity adds a layer of complexity to its production and highlights the importance of efficient usage and recycling in industrial applications.

The discovery of effective methods to trap and separate Xe from other gases is crucial. Xe is also produced in nuclear reactions from uranium fission, and its accumulation partially contributed to the Chernobyl disaster,^[^ ^12^ ^]^ highlighting the need for advanced Xe management strategies. Consequently, the nuclear energy industry is interested in controlling the release of radioactive isotopes of Xe produced in nuclear reactors.

The interactions between Xe and metal surfaces have been observed and explained.^[^ ^13^ ^]^ Low‐energy electron diffraction studies conducted at cryogenic temperatures have also experimentally demonstrated an on‐top site adsorption preference for Xe adatoms on metal surfaces.^[^ ^14^, ^15^ ^]^

**Figure 1:**
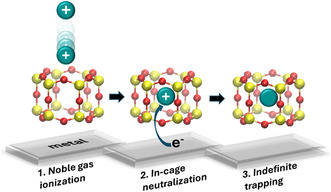
Noble gas trapping mechanism including 1) ionization of noble gas and incorporation in the silicate NC, 2) neutralization by electron transfer from the metal to the gas atom in the NC, and 3) trapping in the NC indefinitely or until thermal release.

Prior studies using crystalline 2D silicate and aluminosilicate materials on Ru(0001) showed that these structures could irreversibly trap argon gas.^[^ ^16^ ^]^ The trapping occurred within hexagonal prism‐shaped aluminosilicate nanocages  (with a unit cell and cage size of ≈0.55 nm, in registry with the unit cell of Ru(0001)). In the case of silicates, trapping occurred both within the NC and at the interface with the metal support. Further work showed that all noble gases larger than neon can be trapped at room temperature through a mechanism in which X‐rays from a synchrotron light source ionized the gas atoms, followed by these ions entering the cages and getting neutralized within them by electron transfer from the metal.^[^ ^17^ ^]^ The noble gas atoms remained in the cages and could be removed by heating to elevated temperatures, that is, Ar: 348 K, Kr: 498 K, Xe: 673 K, and Rn: 775 K.^[^ ^17^, ^18^ ^]^ These two references contain, in addition to the experimental work, theoretical calculations using ab‐initio methods describing the energetics of trapping and releasing the noble gases from the NC. The method for trapping noble gases by ionizing and neutralizing them within a metal‐supported cage was recently patented.^[^ ^19^ ^]^ The mechanism is illustrated in **Figure** **1**. Note that this method differs from ion implantation, in which the ions are accelerated toward a surface. The latter is a very interesting and highly studied area, including the cases of noble gas ions implanted under 2D materials such as graphene and boron nitride.^[^ ^20^, ^21^, ^22^, ^23^ ^]^ In the case described in this article, the noble gases are ionized, but they are not accelerated toward the surface.

While trapping noble gases can be promising for various applications, as alluded to in the introduction section, synthesizing such 2D silicates is very expensive, time‐consuming, and leads to surface areas of just 1 cm^2^, which is impractical.^[^ ^24^ ^]^ Additionally, prior work required using a synchrotron light source to generate enough flux of X‐rays to ionize and trap the gases. More recent work showed an alternative structure, Dodecaphenyl‐polyhedral oligomeric silsesquioxane (DP‐POSS), deposited on a metal film, which resulted in successful Xe trapping after a series of post‐treatments of the material.^[^ ^25^ ^]^ DP‐POSS molecules have the same hexagonal prism cage structure, that is, the building block of 2D materials described above. Note that this material is less well defined than crystalline silicate versions in previous work. While that work resulted in a more straightforward way to obtain silicate NC, it still required a synchrotron to ionize the gas, the material was only 1 cm^2^, and it used a precious metal (Ru) as a support.

**Figure 2:**
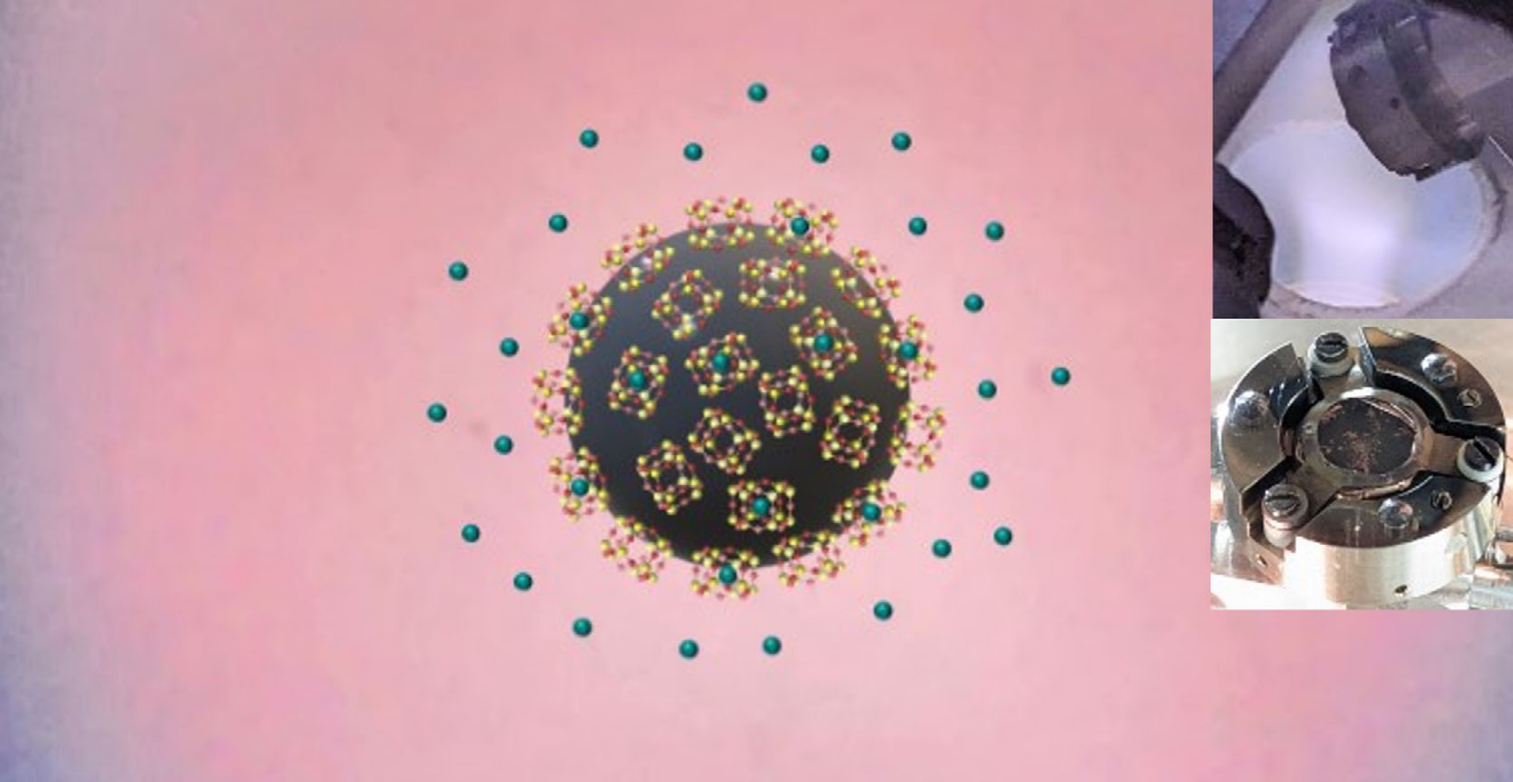
Illustration of a metal particle from the powder covered with hexagonal prism silicate NC in the presence of Xe plasma. The insets show photos of the sample in the center of a sample holder (bottom right) and the sample exposed to Xe plasma (top right).

In the work presented here, we describe two methods to produce high‐surface area materials with NC, using a nonprecious metal Co in addition to Ru, and ionizing the gas without needing a synchrotron, using a plasma. An illustration of a metal particle covered with NC in the presence of plasma is shown in **Figure** **2**. Photos of the sample mounted in a holder and a photo of the sample facing a plasma during one of the experiments are shown as insets. The significant advances described in this work position this functional nanotechnology much closer to applications. This can have significant implications in various fields ranging from clean energy to health, waste removal, and space travel, among many others.

## 2 Results

Two different methods were used to deposit DP‐POSS NC on the surface of Ru and Co powders. One method is chemical vapor deposition (CVD) of DP‐POSS, and the second one is wet impregnation (WI) with a DP‐POSS solution in toluene. Both methods were followed by a series of postprocessing procedures, including annealing in air to burn the organic ligands of the NC precursor and H_2_ annealing to reduce the metal component. The details are described in the Experimental Section. In the remainder of the manuscript, these four samples will be referred as NC‐Ru‐CVD, NC‐Co‐CVD, NC‐Ru‐WI, and NC*‐*Co‐WI. In all cases, the samples are introduced into a high‐vacuum system with a 10^−7^ mbar base pressure, followed by exposure to 0.1 mbar of Xe. The Xe is ignited into a plasma using a copper wire through a feedthrough in front of the sample, using 1 kV AC at 22 kHz (more details in the experimental section).

### 2.1 Xe Trapping in CVD Samples

Both Co and Ru samples were prepared using the CVD method. The XPS results are shown in this section for these samples before and after Xe trapping. In the case of Co, there are additional experimental sets related to samples before and after an additional reduction step in the presence of pure H_2_ at high temperatures in a high‐vacuum chamber.

#### 2.1.1 Xe Trapping in NC‐Co‐CVD Samples

**Figure 3:**
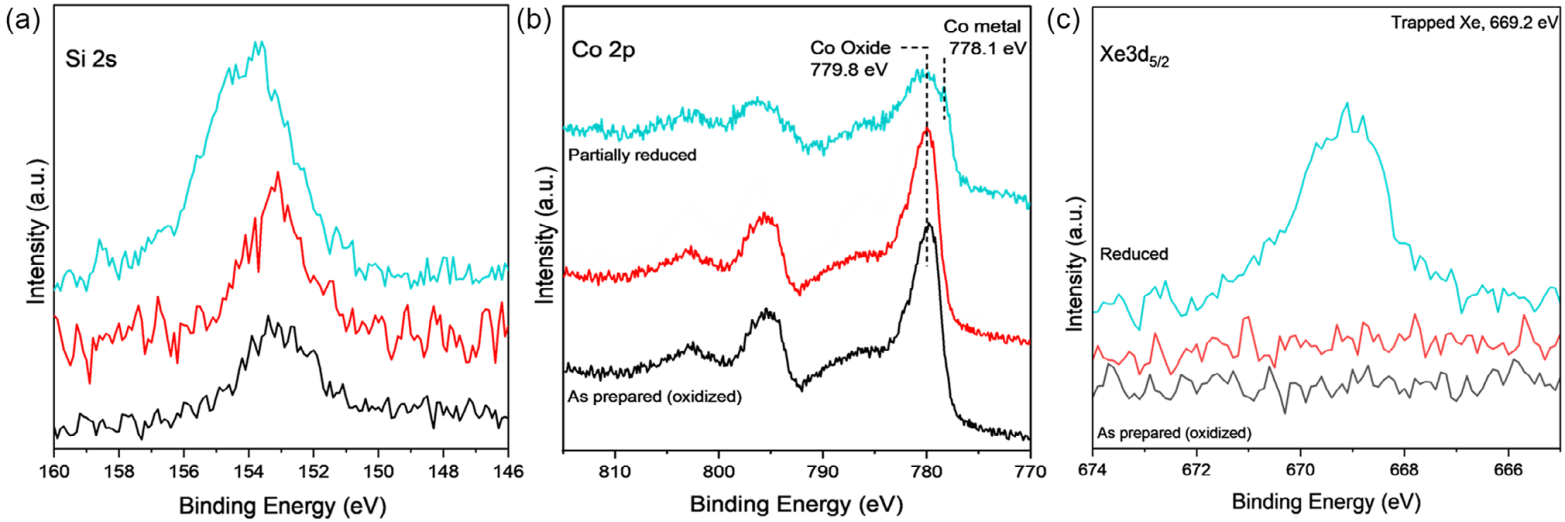
a) Si 2s, b) Co 2p, and c) Xe 3d_5/2_ XP spectra for as‐prepared (black) and after (red‐teal) exposure to Xe plasma, for NC‐Co‐CVD samples. The red spectra were taken after exposure to Xe plasma for as‐prepared sample. The teal spectra correspond to the sample after in situ reduction in H_2_ followed by Xe plasma exposure. All sets of XP spectra, as synthesized and partially reduced, are taken in the UHV system before and after exposure to Xe^+^ plasma for 5 mins.

A sample with NC deposited on Co powder by CVD (NC*‐*Co‐CVD) was analyzed by XPS. **Figure** **3** shows Si 2s, Co 2p, and Xe 3d_5/2_ spectra after sample preparation, after ex situ annealing in Ar/H_2_ in a tube furnace, and pressed in Cu foil in black. Si 2s spectra shows the presence of silicon. The Co 2p and Xe 3d_5/2_ core level spectra show oxidized Co and absence of Xe (as expected since no Xe exposure has occurred yet), respectively. The binding energy of Co 2p_3/2_ at 779.8 eV and a broad shoulder around 785 eV are the characteristics of oxidized Co.^[^ ^26^ ^]^ Details about the NC coverage calculations are included in the Supporting Information but briefly, the Si 2p to Ru 3d peak area ratio of the bilayer structure is used as a reference, and known atomic sensitivity factors are used to allow comparison with other core level spectra of these and other elements. In the case described in this section, the Si 2s and the Co 2p core level spectra (Figures 3a,b) are used for this calculation in comparison to the literature reference to determine a coverage of 1.1 monolayer equivalent (MLE). Here, we define MLE as equivalent to one monolayer of cages covering the surface compared to prior studies of silicate bilayer samples on Ru(0001). Note that what in the literature is called a silicate bilayer corresponds to a monolayer of cages, as it refers to the two layers of silicon atoms that make the 2D structure. As expected, the as‐prepared sample has no Xe (black spectrum in Figure 3c).

The sample is then exposed to 0.1 mbar of Xe plasma for 5 mins at room temperature in a chamber that has a base pressure of 10^−7^ mbar. After evacuating the Xe gas, the sample is transferred to the XPS chamber (within the same ultra‐high vacuum (UHV) system) without exposure to air. XPS spectra of the sample exposed to Xe plasma are shown in red, with no evidence of trapped Xe. While the sample had already been annealed (ex situ) in a reducing environment (4% Ar/H_2_) for 2 h at 500 °C, it still did not show metallic Co . This could be because either these conditions are not enough to reduce the Co oxide or because Co oxidized during the transfer through air into the XPS chamber.^[^ ^27^ ^]^

The sample was then reduced (in situ) by exposure of 0.3 mbar H_2_ at 800 K for 4 h which resulted in the partial reduction of Co as shown in Figure 3b (teal spectra). The sample is then subjected to the same Xe trapping conditions (0.1 mbar Xe plasma for 5 min) and characterized by XPS. The observed XPS peak at 669.2 eV, teal spectrum in Figure 3c, corresponds to the Xe 3d_5/2_ component, showing the successful trapping of Xe gas atoms. The Co 2p core level spectrum in Figure 3b shows a new feature at 778.1 eV, which is assigned to metallic Co.^[^ ^26^ ^]^ Similarly, the O 1s spectra in Figure S1, Supporting Information, show that the component associated with oxidized Co* (CoO_x_) decreased upon H_2_ annealing, supporting that Co is partially reduced. This shows that Co needs to be in a metallic state for the NC to trap Xe gas atoms, as it was suggested previously for flat single crystal samples with a silicate bilayer structure.^[^ ^18^ ^]^ It should be noted that plasma plays a fundamental role in Xe trapping process, as the cations present in the Xe plasma are the ones that are being trapped and then neutralized^[^ ^17^ ^]^ as they enter the cage. As opposed to prior work, which required a synchrotron light source to ionize the gas, making a plasma is a much simpler and cost‐effective way of ionizing the gas. The quantification of Xe trapping capacity is done by comparing the ratio of Si 2p and Xe 3d_5/2_.^[^ ^28^ ^]^ It is estimated that the ≈0.21 Xe atoms are trapped per silica NC (12 Si atoms). The calculations are shown in the supporting information Table S2.

#### 2.1.2 Xe Trapping in NC‐Ru‐CVD Samples

**Figure 4:**
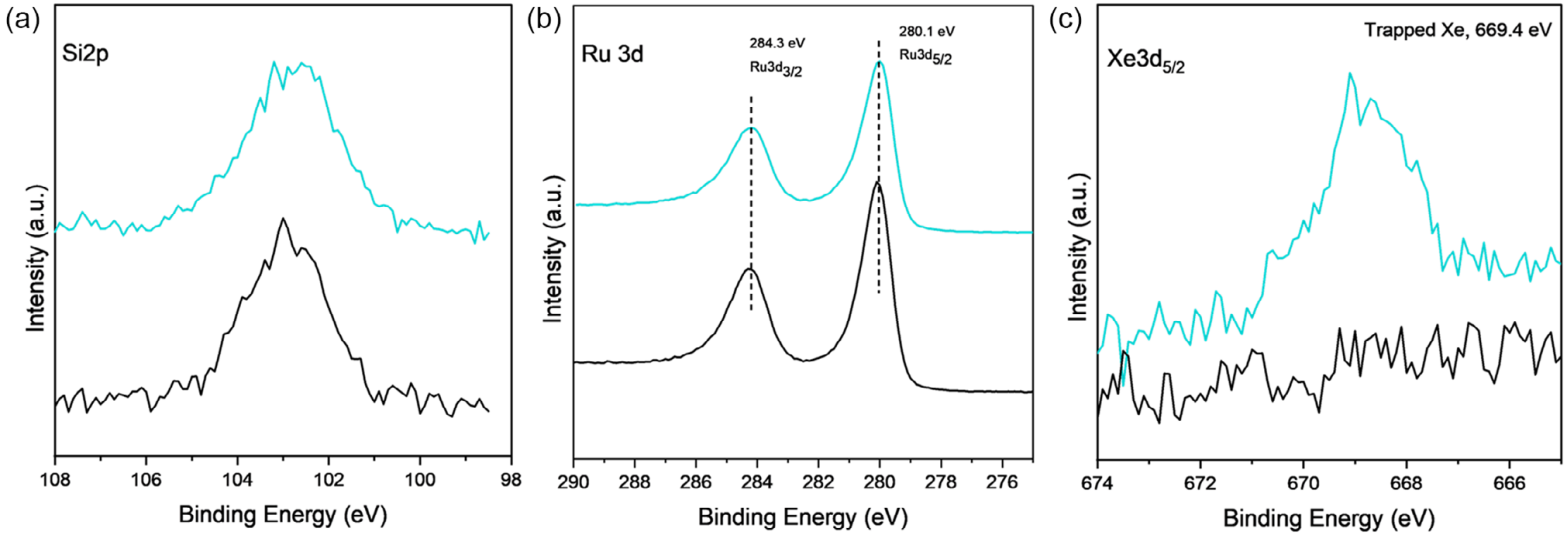
a) Si 2p, b) Ru 3 d, and c) Xe 3d_5/2_ XP spectra for as‐prepared (black) and after (teal) exposure to Xe plasma, for NC‐Ru‐CVD samples. The teal spectra correspond to the sample after in situ reduction in H_2_ followed by Xe plasma exposure. All sets of XP spectra, as synthesized and reduced, are taken in the same UHV system before and after exposure to Xe^+^ plasma for 5 mins.

A NC‐Ru‐CVD sample was prepared following the procedure described in the Experimental Section. **Figure** **4** shows Si 2p, Ru 3d, and Xe 3d_5/2_ core level spectra before and after Xe trapping. The Si 2p in Figure 4a shows a clear indication of the presence of silica cages, from which a coverage of ≈1 MLE is estimated (calculations in Table SI). While the Ru 3d spectrum shows that Ru was in the metallic state (black spectra), the sample was exposed to 0.3 mbar H_2_ at 800 K for 4 h as a precaution. No changes were observed in the Ru 3d spectrum.

The sample was subsequently subjected to Xe plasma (0.1 mbar) at room temperature for a duration of 5 min. Following the removal of the gas from the chamber, XPS spectra showed a peak at 669.4 eV for the Xe 3d_5/2_ core level, attributed to the trapped Xe in the NC. The quantification of Xe coverage based on the intensity ratio of Xe 3d_5/2_ and Si 2p accounts for their atomic sensitivity factors and gives a Xe coverage of 0.35 Xe atoms per silica NC (calculations in the Supporting Information).

### 2.2 Xe Trapping in WI Samples

#### 2.2.1 Xe Trapping in NC‐Co‐WI Samples

The NC*‐*Co‐WI sample was synthesized through the WI method, following the steps described in the experimental section.

**Figure 5:**
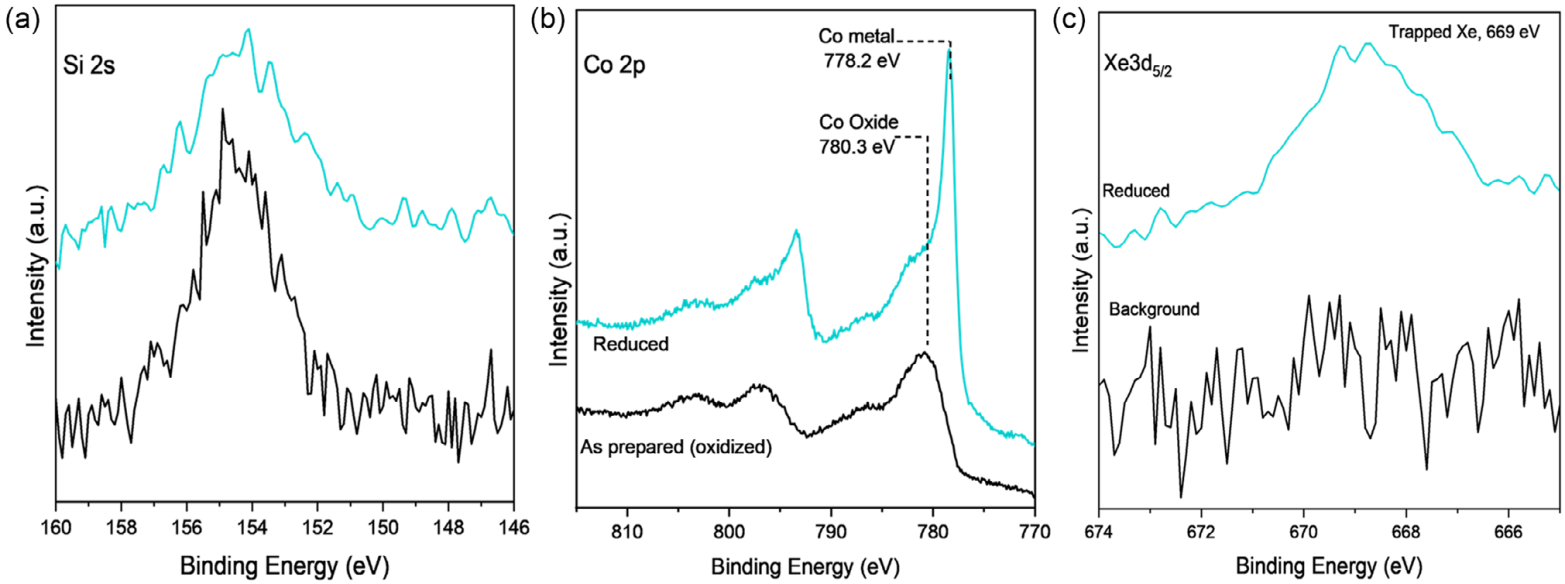
XPS spectra of a) Si 2s, b) Co 2p, and c) Xe 3d_5/2_ core levels taken before (black) and after (teal) the exposure of NC‐Co‐WI samples to 0.3 mbar H_2_ at 800 K for 4 h , followed by 0.1 mbar of Xe plasma at 300 K. All sets of XPS spectra, as synthesized and partially reduced, are taken in the same UHV environment without air exposure in between.

The Si 2s peak observed via XPS, as illustrated in **Figure** **5**a, indicates the presence of silica cages, and an estimated coverage of 1.3 MLE was obtained as described in the supporting information. Since Co oxidizes easily, the sample is reduced (in situ) by exposure to 0.3 mbar H_2_ at 800 K for 4 h. This resulted in a reduction of the intensity of the peak at 780.3 eV from CoO_x_, and appearance of a peak at 778.2 eV (teal spectrum) corresponding to metallic Co, as can be seen in Figure 5b. There, the black spectra correspond to the as‐prepared sample, while the teal spectra correspond to the sample after H_2_ annealing and Xe plasma exposure. The observed XPS peak at 669.0 eV, teal spectrum in Figure 5c, corresponds to the Xe 3d_5/2_ component, which demonstrates the successful trapping of Xe gas atoms in silica cages supported by Co metal. The quantification of the coverage of trapped Xe is similarly done by comparing the Xe 3d_5/2_/Si 2s ratio to the reference silicate bilayer sample. It is estimated that ≈0.2 Xe atoms are trapped per silica NC (12 Si atoms).

#### 2.2.2 Xe Trapping in NC‐Ru‐WI Samples

**Figure 6:**
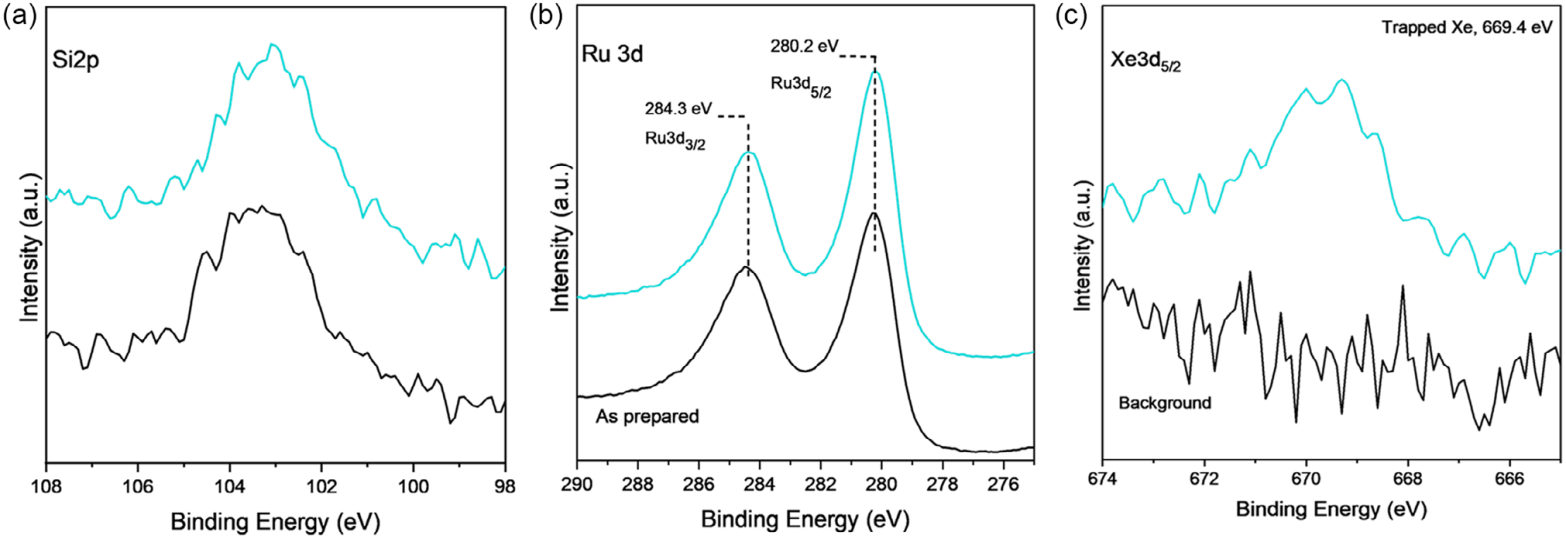
XPS spectra of a) Si 2p, b) Ru 3d, and c) Xe 3d_5/2_ core levels taken before (black) and after (teal) the exposure of NC‐Ru‐WI samples to 0.1 mbar of Xe plasma at 300 K. All sets of XPS spectra are taken in the same UHV environment before and after exposure to Xe^+^ plasma for 5 mins.

Similar experiments were performed on silica cages supported on Ru powder prepared through the WI method. A coverage of ≈1 MLE was determined based on the Si 2p to the Ru 3d_5/2_ peak area ratios. The corresponding Si 2p, Ru 3d, and Xe 3d_5/2_ spectra are shown in **Figure** **6** before and after exposure to Xe plasma. The Ru 3d_5/2_ peak at 280.2 eV (black spectrum in Figure 6b) indicates Ru is metallic for the as‐prepared sample. As for all other cases, the sample was annealed (ex situ) in a reducing environment (4% Ar/H_2_ for 3 h) to reduce RuO_x_ resulting from the calcination step that was used to remove the organic ligands in the POSS molecule.

After the ex situ H_2_ annealing, the NC‐Ru‐WI sample is exposed to 0.1 mbar Xe plasma at room temperature for 5 min. After evacuating the Xe gas from the chamber, the sample surface was characterized by XPS. A peak at 669.4 eV (teal) in Figure 6c is assigned to Xe atoms trapped in the silica NC. The Xe coverage is estimated at ≈0.45 Xe atoms per NC (calculations included in the Supporting Information).

## 3 Discussion

Four different samples with high‐specific surface areas (≈10 m^2^ g^−1^) are explored in this work. Two correspond to silicate NC on Ru and two use Co as a support. The surface area per unit of weight for these samples is approximately five orders of magnitude higher than flat samples reported in related prior work (≈0.0001 m^2^ g^−1^). For each metal, a vapor deposition and a wet impregnation method were tested. The NC precursor used in all cases was DP‐POSS, which, in addition to the hexagonal prism cage unit, it has a phenyl ring linked to each of the 12 silicon atoms in the vertices of the prism. To remove such organic ligands, a calcination step was used to burn these hydrocarbon moieties. It is worth noting that all samples end up with a coverage close to 1 MLE of NC. It is believed that even if we start with higher coverages of NC upon deposition, the annealing step removes the multilayer, always getting close to a coverage of 1 MLE (see **Table** **1**). This is expected, as the interaction between the first and second DP‐POSS layers is expected to be close to that of the bulk material, which is volatile at temperatures lower than the ones used during the calcination step mentioned above. As this calcination step can result in oxidation of the metal support, a subsequent H_2_ annealing step was used. While this step was sufficient to produce samples with metallic Ru, still the cases of Co resulted in an oxidized metal when probed by XPS. Note that prior work on flat samples, and this work for the powder material, shows that the metallic state is required in the subsequent trapping step. Thus, all samples were exposed to H_2_ at much higher temperatures resulting in some of the Co being in the metallic state. These samples did then (after reduction) show successful trapping of Xe. For the case of Co powder, the Xe coverage was ≈0.2 atoms per NC for both vapor deposition and WI methods. For the case of Ru powder as support, the Xe coverage was 0.35 and 0.45 Xe atoms per cage for vapor deposition and WI methods, respectively. The higher coverage in the case of Ru could be related to being fully reduced while Co was still partially oxidized, or it could be related to some other inherent chemical role the metal may play in the trapping. Note that prior work on flat systems showed interesting behavior indicating potential covalent interactions between Ru and Xe at the interface between the NC and the metal support.^[^ ^1^ ^]^ While this cannot be directly assessed from this work, there is a significant possibility that the nature of the metal is important beyond its ability to remain in the metallic state and this deserves further investigation. We also note that while we expect most of the population of trapped Xe to be inside the cages, prior work on flat systems has shown that a fraction of the atoms can be trapped at the interface with the metal. In any way, the fact that such an inexpensive metal as Co (over 700 times cheaper than Ru as of February 2025) works as a support can be significant for many applications in which cost is a critical factor.

**Table 1:** Summary of NC coverage (*θ*_NC_) and Xe (*θ*_Xe_) coverage for each of the samples.

Sample	*θ*_NC_	*θ*_Xe_
NC*‐*Co‐CVD	1.1	0.21
NC‐Ru‐CVD	1	0.35
NC*‐*Co‐WI	1.3	0.2
NC‐Ru‐WI	1	0.45

Another important aspect of this work is the ionization method used, which involves producing a cold plasma ignited by a 1 kV AC at 22 kHz applied through a feedthrough in the experimental vessel. This allows experiments in simple setups in contrast to prior experiments that required X‐rays from a synchrotron light source as a source of ionization. In addition to simplifying experiments, this method shows promise to practical applications as ionizers that rely on applying voltage are common in a variety of devices. We want to emphasize as well that, while ions in the plasma do have a kinetic energy potentially higher than that given by temperature, this is not sufficient to produce any kind of implantation. This is evident by the fact that Xe is not trapped in the cages in the presence of plasma when the metal is oxidized and this is consistent with prior work on flat systems.^[^ ^18^ ^]^

While in prior synchrotron‐based experiments, the X‐rays were the ones responsible for ionizing noble gases during ambient‐pressure XPS (AP‐XPS) experiments, and this contributed a vast amount of fundamental knowledge, this is far from being a practical way of producing ions. Note as well that, while the experiments in this work used a laboratory‐based X‐Ray source, and a lab‐based AP‐XPS system could have been used in principle, the photon flux generated is insufficient to ionize enough Xe atoms.

## 4 Conclusions

This work showcases three major advances toward developing a material that can trap noble gases for practical applications. These are: 1) synthesis of high‐surface area materials, 2) use of less expensive metal supports, and 3) use of a simple ionization method, that does not require the use of a synchrotron light source. To do this, commercially available precursors that have a hexagonal prism‐shaped silicate nanocage structure were used as building blocks and deposited onto Co or Ru powders. This resulted in trapping materials with surface areas in the order 10 m^2^ g^−1^—much larger than samples in prior work, which were limited to 1 cm^2^. The addition of Co as an option demonstrated that cheaper metals can be used successfully (only Ru was used before), but with the downside that they oxidize more easily. As a way to produce Xe ions, a cold plasma was ignited in the sample environment, leading to similar trapped Xe coverages (0.1–0.5 atoms per cage) as prior work on flat samples which had used a high flux of X‐rays at a synchrotron light source. These advances both in the fundamental understanding of the trapping process, and in simplifying the synthesis and ionization methods, bring this technology much closer to practical applications.

## 5 Experimental Section

5.1

5.1.1

##### Sample Preparation Methods: CVD

**Scheme 1:**
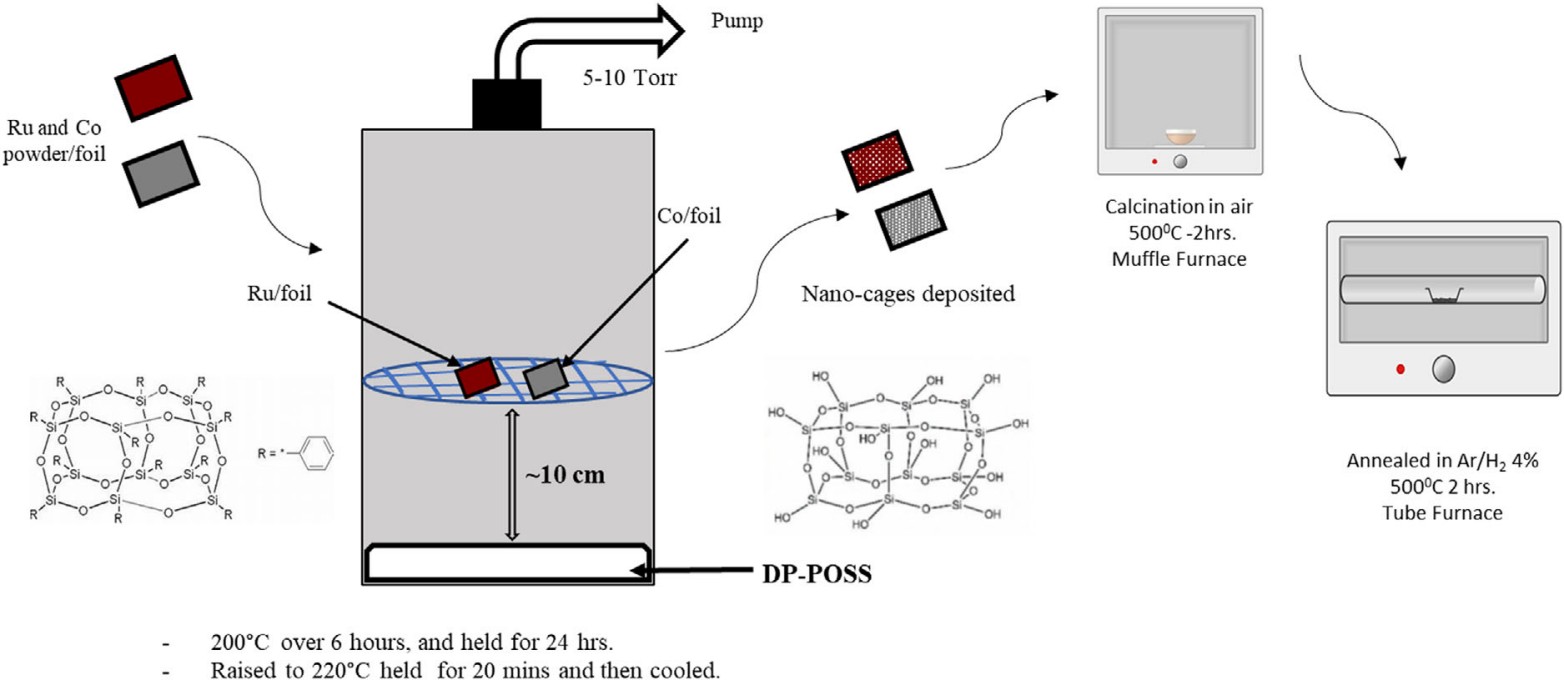
Schematic representation of the approach to deposit silica NC on the Ru and Co metal powder using CVD technique. The deposited cages are calcined and annealed after the deposition to remove the organic components.

DP‐POSS molecules were deposited onto Ru and Co metal powders using the CVD technique (**Scheme** **1**) at Forge Nano Inc. The source material for silica NC and DP‐POSS was purchased from Sigma‐Aldrich (CAS: 18 923‐59‐6). Metal powders of Co (CAS: 7440‐48‐4) and Ru (99.9%, APS: 0.5–1 μm) were purchased from Sigma Aldrich. Ru and Co powders in as‐received condition were first pressed on 1 mm‐thick Cu foil and sent to Forge Nano for deposition of DP‐POSS as described below.

The deposition of silica cages was done by placing two foils individually pressed with metal powder of Ru and Co in a sealed vessel with 0.1 g of DP‐POSS (solid) placed in the bottom of the vessel (Scheme 1). A 60‐micron filter was placed on the outlet side of the vessel to prevent powder from escaping. The vessel was evacuated to 5 Torr and then sealed off.

The vessel was then heated to 200 °C, over a ramp time of 6 h, and held at this temperature for 24 h, followed by raising the temperature to 220 °C for another 20 min. The vessel was then cooled to room temperature and samples were removed. The samples were then calcined at 500 °C in muffle furnace, followed by the reducing/annealing in Ar/H_2_ (4%) for 2 h at the same temperature. The prepared samples were then mounted on the sample holder for further characterization and Xe trapping experiments.

##### Sample Preparation Methods: WI

**Scheme 2:**
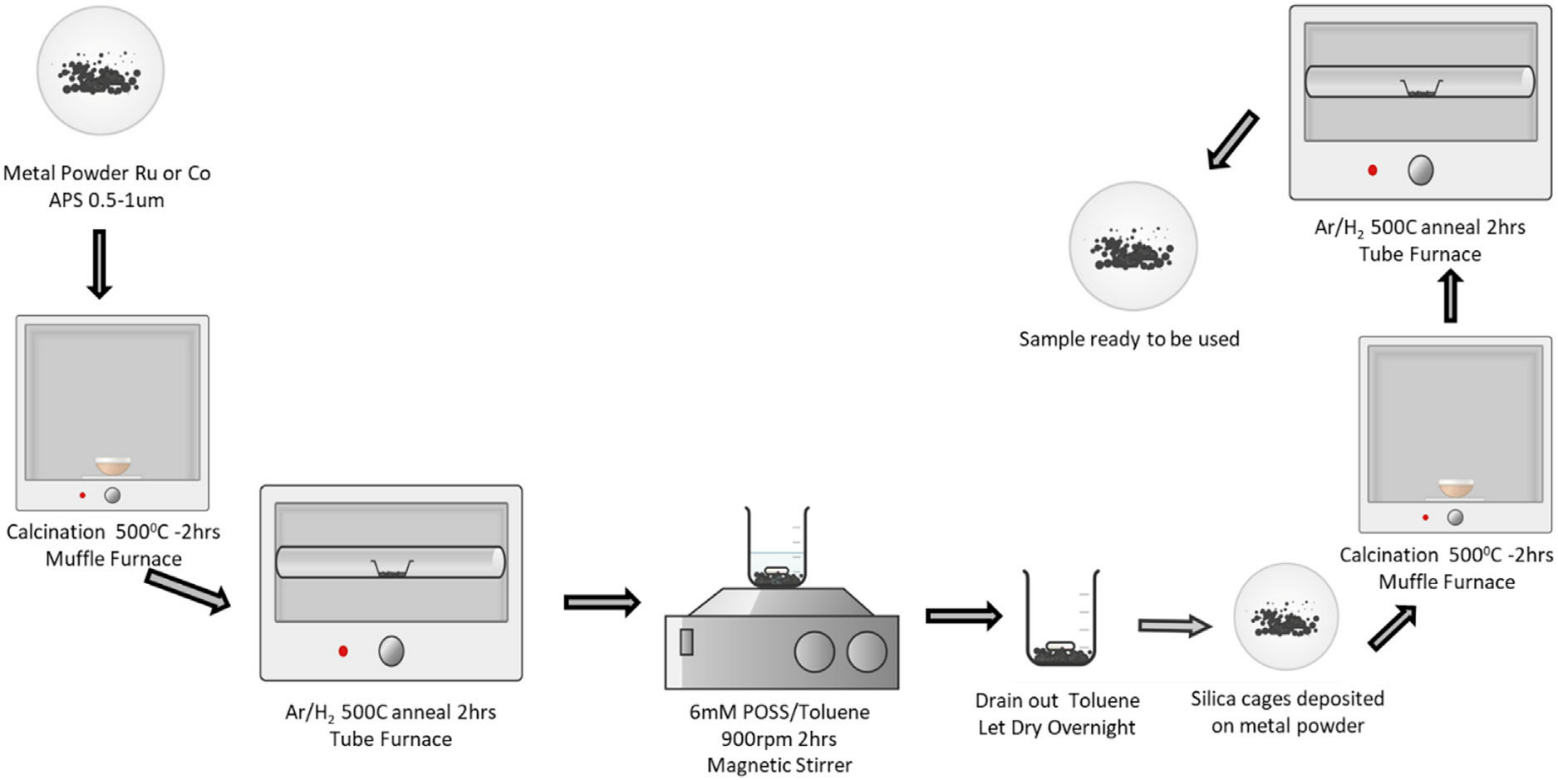
Schematic representation of the approach to deposit silica NC on Ru and Co metal powder using WI method.

Metal powders of Ru and Co were first calcined in the air for 2 h at 500 °C followed by reducing/annealing in Ar/H_2_ (4%) for 2 h at 500 °C. The schematic representation for preparation of silica cages on Ru or Co powder is shown in **Scheme** **2**.

54 mg of D‐POSS was dissolved in 6 ml of toluene, preparing a 6 mM solution; 150 mg of pre‐treated Ru or Co powder was added to the solution and mixed with the help of a magnetic stirrer for ≈2 h (900 rpm). After mixing, the metal powder was left to precipitate, the solution was drained, and the powder was dried. The obtained dry sample was again subjected to calcination for 2 hr at 500 °C to burn off the organic ligands present in the DP‐POSS molecule. Lastly, the sample was annealed at 500 °C in a reducing environment (Ar/H_2_ 4%) for 2 h. Once the silica/metal powder was prepared, it was pressed on copper foil using a hydraulic press with a pressure of 1 Ton for 1 min.

Scanning electron microscopy (SEM) images documenting the Ru and Co samples prepared through the WI method, before and after the deposition of NC, were included in the supporting document (Section S2, Figure S2, Supporting Information).

In both cases, before and after deposition revealed a consistent and unaltered morphology, suggesting the absence of substantial structural changes when observed at a microscopic scale of 1 μm and a working distance of ≈5 mm.

Gas adsorption isotherms were measured at 77 K using a Micromeritics 3flex gas analyzer in order to determine the surface area of the NC‐Ru‐WI and NC*‐*Co‐WI powders. Due to small sample sizes, krypton (Kr) was chosen as the adsorbate due to its improved accuracy for low surface areas. The surface areas of the NC‐Ru‐WI and NC‐Co‐WI powders were determined using the Brunauer–Emmett–Teller model in the range P/P_0_ = 0.05–0.3. Prior to analysis, all samples were degassed under vacuum at 100 °C until an ultimate pressure of 10^−4^ torr was reached in order to remove adsorbed moisture. The analysis showed that the surface area was 9.49 m^2^ g^−1^ for Ru with silica NC and 11.33 m^2^ g^−1^ for Co with silica NC. These results demonstrated significantly larger surface area for metal powders when compared to thin films, underscoring the primary motivation for scaling up from thin films to powders in this study (Table S3, Supporting Information).

## Conflict of Interest

The authors declare no conflict of interest.

## Supporting information

Supplementary Material

## Data Availability

The data that support the findings of this study are available from the corresponding author upon reasonable request.
