# Supplementary material for: Trapping Xe in Nanocages Using a Plasma
Source: Small Sci. 2025 Jul 11;5(10):2500136. doi: 10.1002/smsc.202500136 (PMC12499482; doi:10.1002/smsc.202500136)
Supplement: Supplementary file 1 — Supplementary Material [file SMSC-5-2500136-s001.pdf]

Trapping Xe in nanocages using a plasma

*Laiba Bilal, Asim Khaniya, Dustin Olson, Staci Moulton, Arrelaine Dameron, Xiao Tong, Lynne Ecker, Dario Stacchiola, Jorge Anibal Boscoboinik**

Section S1: Calculations of nanocage coverage and Xenon atoms per silica nanocage

The coverage of nanocages is estimated to be in units of monolayer equivalents (MLE). In this paper a MLE is considered as the equivalent to the total coverage of a silicate bilayer structure ("a monolayer of cages") on Ru(0001).[1] In this structure, two hexagonal silicate layers connected by oxygen atoms make a 2D-structure of interconnected hexagonal prism cages that perfectly tiles the surface.

The Si 2p/Ru 3d peak area ratio is compared to that of an experimentally grown silica bilayer on Ru(0001). The latter is experimentally measured to be approximately 0.025[2] when using an Al k-α X-ray source. An equivalent conversion factor is obtained by Co, by considering the ratio of atomic sensitivity factors (ASF) of the Co 2p and Ru 3d core levels. These empirical ASF for each core level allow the quantitative comparison between different elements. All values of ASF are taken from the appendix[3] of Handbook of X-ray Photoelectron Spectroscopy by John F. Moulder and William F. Stickle[4] for X-ray source at 54.7°.[3] The peak area for Si 2p for Co-CVD is calculated from its Si 2s peak area. (Si 2p = 0.95×Si 2s) [5]

Table S1. The table shows the values of peak area for Si 2p, Ru 3d and Co 2p for all four samples and their calculated coverage of cages in units of MLE (1 MLE is one full monolayer of cages in the bilayer silicate).

Sample	Peak area		
	Si 2p	Ru3d/Co2p	MLE
Co-CVD	2545.05	77721	1.1
Ru-CVD	1396	56967	1
Co-WI	1418.35	36525	1.3
Ru-WI	1766	71940	1

To find the coverage of xenon atoms per nanocage, we use the peak area ratio of Xe $3d_{5/2}$ and Si 2p and correct them by using experimental ASFs, (dividing the pk area by its ASF).[3] The calculated ratio is then multiplied by 12 since to account for the 12 Si atoms making up a single cage.

$$\text{Xe atoms per nanocage} = \left\{ \frac{\text{Area of Xe } 3d_{5/2} \text{ (ASF corrected)}}{\text{Area of Si2p (ASF corrected)}} \right\} \times 12$$

Table S2. The table shows the values of peak area for Xe $3d_{5/2}$, Si 2p and the number of Xe atoms calculated per NC using the above equation.

Sample	Peak area		
	Xe $3d_{5/2}$	Si 2p	Xe atom/NC
Co-CVD	673	3017	0.21
Ru-CVD	673	1187	0.35
Co-WI	363	1418.35	0.2
Ru-WI	1278	1753	0.45

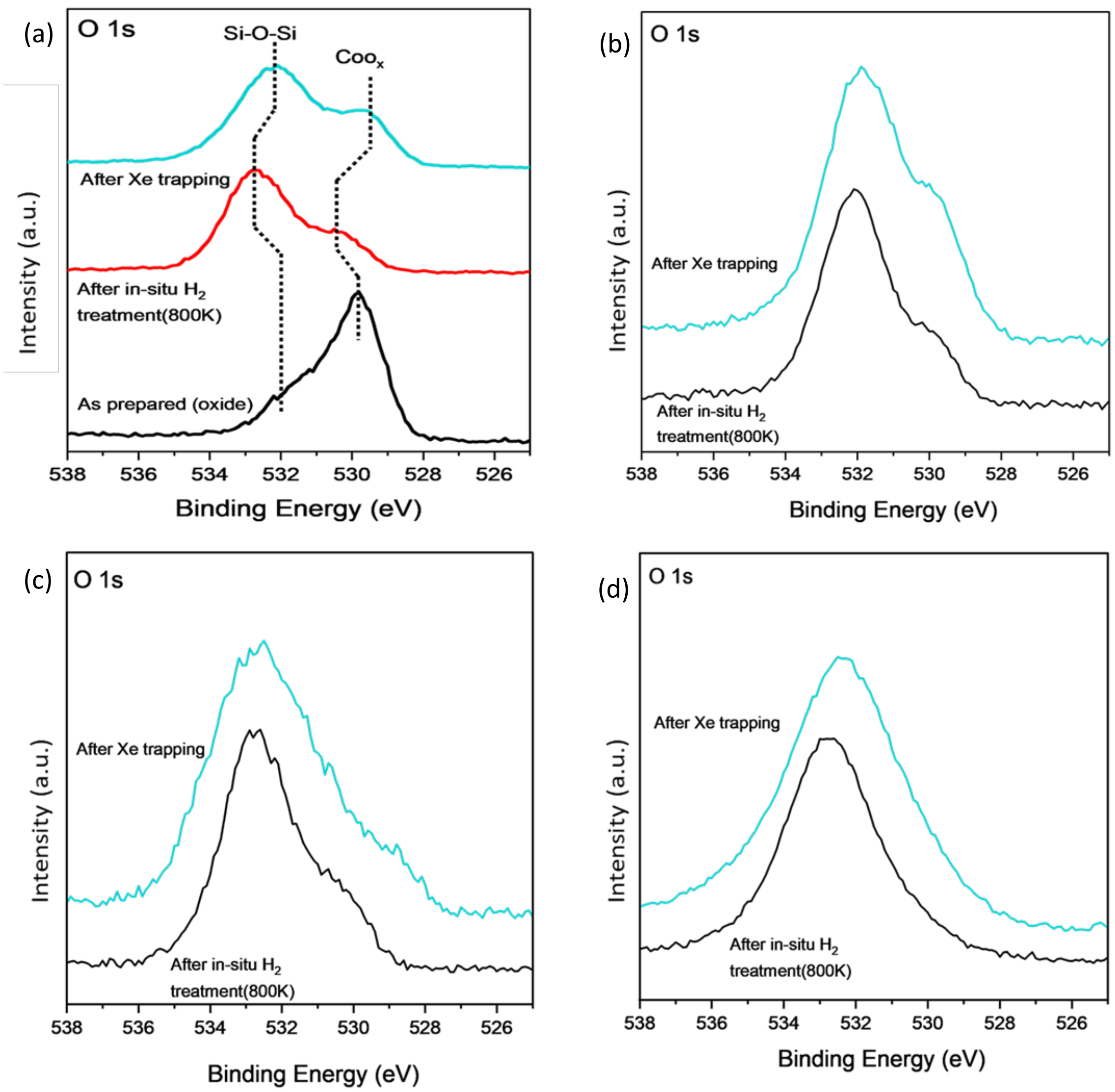

Figure S1. (a) The O 1s spectra for Co-CVD shows that the O component associated with CoO_x decreases upon H_2 annealing, indicating that Co is partially reduced. **(b)** O 1s spectra for Ru-CVD **(c)** O 1s spectra for Co-WI **(d)** O 1s spectra for Ru-WI

Section S2. SEM Characterization for samples prepared by WI

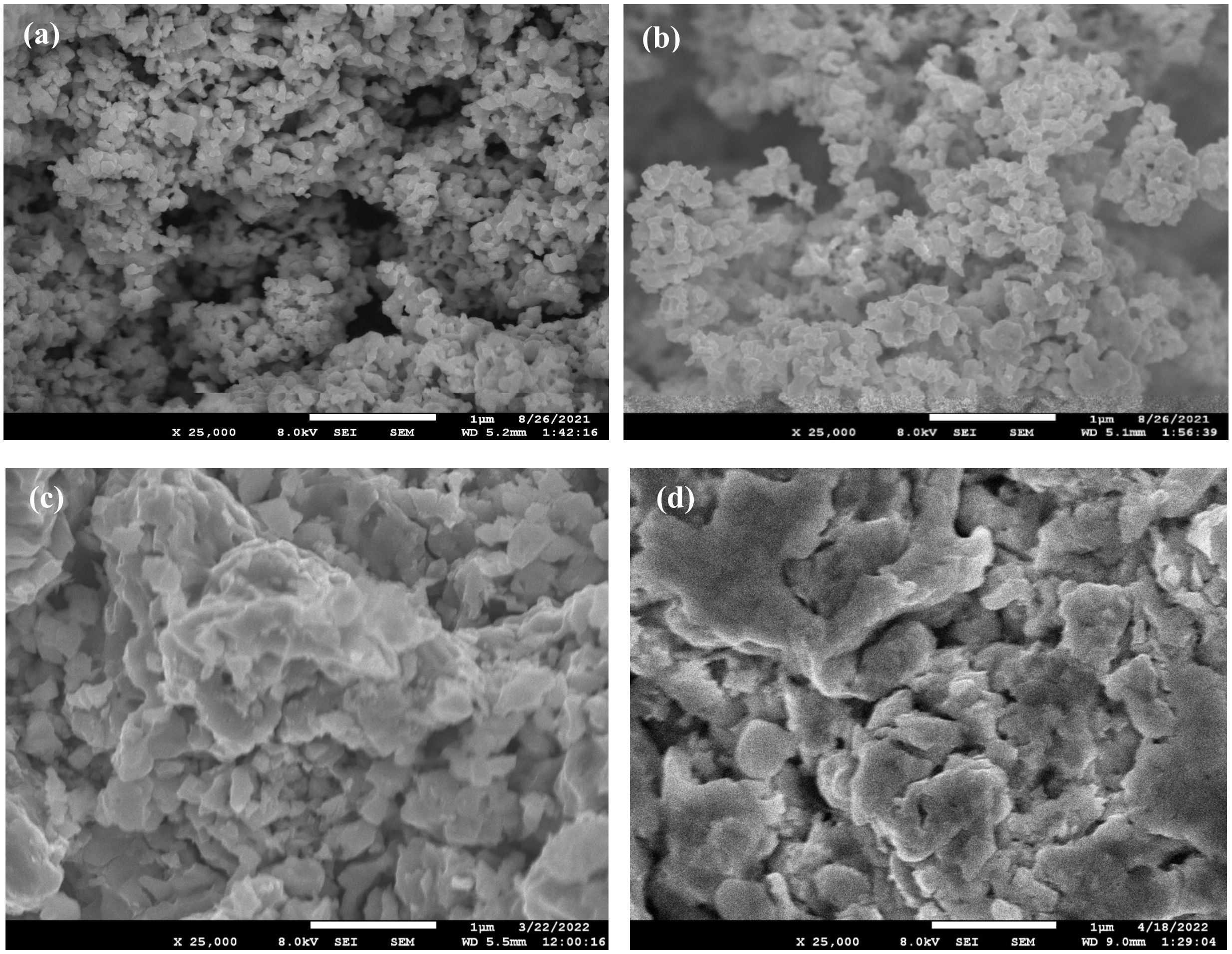

Figure S2. Images of Ru (top) and Co (bottom) metal powders before and after silica cage deposition via wet impregnation method. (a) Ru, (b) SiO_2/Ru (c) Co (d) SiO_2/Co

Section S3: BET results for samples prepared by WI

Brunauer–Emmett–Teller (BET) surface area analysis was performed using krypton (Kr) as the adsorbate gas. The BET method is a standard technique for determining the specific surface area of materials by measuring the amount of gas physically adsorbed onto the surface. Krypton was chosen due to its higher sensitivity at low surface areas, making it suitable for metal powders with relatively low porosity.

Table S3. BET analysis for Ru and Co metal powder with NC

**Ru Powder w/Cages:**	**Run 1**	**Run 2**	**Avg**	
mass : 78.1 mg<br>mass post degas : 52.1 mg	9.4435	9.5456	9.4946	m^2/g
**Co Powder w/Cages:**	**Run 1**	**Run 2**	**Avg**	
mass : 83.5mg<br>mass post degas : 51.5mg	11.0639	11.6054	11.3347	m^2/g

[1] D. Löffler, J. J. Uhlrich, M. Baron, B. Yang, X. Yu, L. Lichtenstein, L. Heinke, C. Büchner, M. Heyde, S. Shaikhutdinov, H. J. Freund, R. Włodarczyk, M. Sierka, J. Sauer, *Phys Rev Lett* **2010**, *105*, DOI 10.1103/PhysRevLett.105.146104.

[2] B. Yang, W. E. Kaden, X. Yu, J. A. Boscoboinik, Y. Martynova, L. Lichtenstein, M. Heyde, M. Sterrer, R. Włodarczyk, M. Sierka, J. Sauer, S. Shaikhutdinov, H. J. Freund, *Physical Chemistry Chemical Physics* **2012**, *14*, 11344.

[3] C. D. Wagner, L. E. Davis, M. V. Zeller, J. A. Taylor, R. H. Raymond, L. H. Gale, *Surface and Interface Analysis* **1981**, *3*, DOI 10.1002/sia.740030506.

[4] J. F. Moulder, W. F. Stickle, P. E. Sobol and K. D. Bomben, Handbook of X-Ray Photoelectron Spectroscopy, Physical Electronics Division, Perkin-Elmer Corp., Norwalk, **1995**.

[5] Casa Software, "CasaXPS Manual 2.3.15 Rev 1.2," **2009**.
